# Differences in Work Disability Duration for Immigrants and Canadian-Born Workers in British Columbia, Canada

**DOI:** 10.3390/ijerph182211794

**Published:** 2021-11-10

**Authors:** Sonja Senthanar, Mieke Koehoorn, Lillian Tamburic, Stephanie Premji, Ute Bültmann, Christopher B. McLeod

**Affiliations:** 1 School of Population and Public Health, University of British Columbia, Vancouver, BC V6T 1Z3, Canada; sonja.senthanar@ubc.ca (S.S.); lillian.tamburic@ubc.ca (L.T.); chris.mcleod@ubc.ca (C.B.M.); 2 School of Labour Studies, McMaster University, Hamilton, ON L8S 4M4, Canada; spremji@mcmaster.ca; 3 Department of Health Sciences, Community and Occupational Medicine, University Medical Center Groningen, University of Groningen, 9712 CP Groningen, The Netherlands; u.bultmann@umcg.nl

**Keywords:** work disability, immigrant workers, workers’ compensation, sex/gender, health equity

## Abstract

This study aimed to investigate differences in work disability duration among immigrants (categorized as economic, family member or refugee/other classification upon arrival to Canada) compared to Canadian-born workers with a work-related injury in British Columbia. Immigrants and Canadian-born workers were identified from linked immigration records with workers’ compensation claims for work-related back strain, connective tissue, concussion and fracture injuries requiring at least one paid day of work disability benefits between 2009 to 2015. Quantile regression investigated the relationship between immigration classification and predicted work disability days (defined from injury date to end of compensation claim, up to 365 days) and modeled at the 25th, 50th and 75th percentile of the distribution of the disability days. With a few exceptions, immigrants experienced greater predicted disability days compared to Canadian-born workers within the same injury cohort. The largest differences were observed for family and refugee/other immigrant classification workers, and, in particular, for women within these classifications, compared to Canadian-born workers. For example, at the 50th percentile of the distribution of disability days, we observed a difference of 34.1 days longer for refugee/other women in the concussion cohort and a difference of 27.5 days longer for family classification women in the fracture cohort. Economic immigrants had comparable disability days with Canadian-born workers, especially at the 25th and 50th percentiles of the distribution. Immigrant workers’ longer disability durations may be a result of more severe injuries or challenges navigating the workers’ compensation system with delays in seeking disability benefits and rehabilitation services. Differences by immigrant classification speak to vulnerabilities or inequities upon arrival in Canada that persist after entry to the workforce and warrant further investigation for early mitigation strategies.

## 1. Introduction

Immigrants represent approximately 28 percent of the workforce in the province of British Columbia, Canada [1], and the proportion of immigrant workers is growing, particularly at younger ages [2]. However, jobs available to immigrant workers are often known as the 3-Ds—dirty, dangerous and demanding—and reflect a pattern of immigrant workers being constrained in their labor market choice and restricted to hazardous jobs [3]. As a result of more hazardous working conditions and environments, immigrant workers have an increased risk of work-related injuries, illnesses and disability compared with native-born workers. For instance, in a review of immigrants’ occupational injuries globally, Salminen and colleagues found that immigrant workers experienced work-related injuries twice as often as native-born workers in part because of their adverse working conditions [4]. More recently, Sterud and colleagues, in their updated review of the literature, also found increased work-related injuries among immigrants, as well as a higher risk of sick leave, attributable to occupational factors [5]. Finally, in a recent study of work disability, defined as disability caused by exposure or conditions at work resulting in time off, immigrants in British Columbia had longer work disability of 3 to 5 days on average following a work injury compared to Canadian-born workers for the same injury [6].

Existing frameworks on immigrants’ work-related health inequalities point to multiple factors that may lead to work-related injury and prolonged work disability duration [7,8]. In addition to working in more hazardous conditions as highlighted above, a lack of language proficiency in English or French as the two official languages in Canada, for example, make it difficult for immigrants to understand occupational health and safety regulations or communicate and report hazards in the workplace [9,10]. Formal occupational health and safety training to protect workers may be inadequate for the level of risk involved with the work [11], not offered in a worker’s native languages [12,13] or, in many cases, not offered at all [9,10,14]. Newness to a job often means immigrants are unfamiliar with their rights to income replacement benefits following a work injury, and this unfamiliarity is exploited by employers seeking cheap labour [14,15,16,17,18]. Studies have found that employers may downplay reported injuries to dissuade immigrants from filing a workers’ compensation claim or to suppress a workers’ compensation claim (e.g., by not filing the employer report) [17,19,20,21], and that immigrants are less likely to resist claim suppression for fear of employer reprisal or other negative consequences [14,22]. Consequently, immigrants may continue to work through an injury that can delay health care treatment and rehabilitation, resulting in longer work disability, and ultimately affect long-term engagement in the labour market [16,23].

### 1.1. Work Disability and Immigration

Empirical evidence on immigrant workers’ experiences after injury in terms of work disability is limited, with a few exceptions [6,24,25,26,27,28]. Work disability as an outcome of interest is important given the effects on a worker’s health, labour market participation and financial security, but also broadly, on policies and programmes to reduce work disability, including workers’ compensation systems and health care services. In a study of Ontario workers’ compensation claimants, for instance, workers with active claims beyond 12 months were more likely to experience financial difficulties when absent from work, poorer self-rated health and greater pain that interfered with normal activities when compared with workers with shorter claim durations (<12 months). These workers were also less likely to be employed, highlighting the adverse consequences of prolonged work disability [29].

Within the context of immigration, work disability is complicated by many of the aforementioned factors (language, newness to a job, occupational health and safety training) that may prevent injury reporting. However, immigrants’ disadvantages may also persist following the filing of a workers’ compensation claim. Administrative burdens associated with social programs, such as workers’ compensation, for example, can have health-harming effects [30]. Immigrants have expressed difficulties navigating the bureaucracy of workers’ compensation, including problems with paperwork, deadlines and lack of timely and appropriate care from health care providers during the adjudication process [14,31]. In the presence of language barriers, some evidence has also found that workers’ language needs were not systematically assessed and addressed by workers’ compensation which led to complexities in the claims process that postponed diagnosis, treatment, rehabilitation and recovery [32,33]. These issues are likely tied to the fact that immigrants have less social agency and/or are unable to exercise their rights and freedoms in the same way as non-immigrants [16] and suggests the need to consider the unique social and economic contexts that shape the work disability experiences of immigrant workers.

### 1.2. Immigration Classification on Arrival to Canada

Immigrants to Canada arrive under three main categories that are associated with different educational, language and employment expectations that may differentially affect their entry into the workforce, working environments and the risk of injury, and disability duration following a work-related injury or illness due to injury severity or delayed care-seeking:

Economic immigrants are individuals with the skills and experience that are needed in the workforce. This group of immigrants comprises skilled workers and business immigrants, including investors, entrepreneurs and self-employed persons. They possess the human (language, education, work experience) and social capital needed to economically integrate into the workforce and often arrive to a job that is not categorized by the 3Ds.

Family member immigrants are individuals who are sponsored to the country by family members in Canada and include spouses and partners (common-law), parents, grandparents and ‘other’ family (dependent children, brothers, sisters, nephews, nieces, grandchildren, etc.). The sponsor is required to provide all financial supports upon arrival, but family member immigrants are eligible to work in Canada when they become permanent residents.

Refugee/other classification immigrants are individuals who have fled their country due to severe hardships, such as war and political turmoil, and seek protection in a host country. The rushed nature of their departure does not afford them the opportunity to prepare for their transition to Canada, and they are selected based on vulnerability rather than human and social capital. Refugee/other classification immigrants are eligible to work in Canada upon receipt of a work permit and social insurance number.

The body of work that has examined outcomes by immigration classification has focused on earnings and employment integration. Studies have consistently found that economic immigrants experience under- and unemployment but that these rates are lower than other immigrant groups [34,35], have higher income earnings along a gradient of time compared with other immigrant groups [36,37,38], and that refugees, in particular, are over-represented in riskier jobs [39]. Economic immigrants’ relative success compared to other immigrant groups is partially attributed to fewer labour market barriers. For example, they are more likely to have their credentials recognized, arrive knowing at least one of Canada’s official languages, and have friends and family that facilitate labour market integration in jobs that are commensurate with their skills [40,41,42]. Given their human and social capital [43], they are less likely to work in hazardous environments, to be aware of their rights, and to navigate health care and insurance systems.

Taken together, these studies suggest that immigration classification is an important conditioning factor to economic integration and, by extension, quality of employment and risk of work injury. More specifically, immigration classification is a global measure of vulnerability and inequity at the time of arrival to Canada and can be considered a surrogate measure with face validity for differential contexts and social determinants of health. Unfortunately, this measure has yet to be examined in the work disability literature. Thus, this study aimed to address the following evidence need for decision- and policy-makers: Does the duration of work disability differ by immigration classification for workers with a work-related injury in the jurisdiction of British Columbia, Canada?

Given the evidence to date, we hypothesize that immigrants and, in particular, family member and refugee/other immigrant classification workers, regardless of when they immigrated to Canada, will have longer work disability experiences than Canadian-born workers. This inequity is due to a combination of more severe injuries and delayed disability and health benefit seeking. Conversely, we hypothesize that economic immigrants’ context of higher human and social capital translates to shorter work disability experiences compared to family member and refugee/other classifications, but longer disability duration compared to Canadian-born workers. The current study offers a novel contribution to the scientific literature with access to workers’ compensation data linked to immigration records for the working population of British Columbia over a longitudinal follow-up period, and detailed immigration classification data as a surrogate measure for the differential contexts and social determinants of health for immigrants upon arrival to Canada that may persist over time. Evidence of differential experiences and long-term consequences for immigrant workers is an essential input to ongoing discourse and to providing a body of evidence for change and action.

## 2. Materials and Methods

### 2.1. Data Sources, Jurisdictional Context and Study Sample

This study used individual-level, linked administrative data provided by WorkSafeBC, the workers’ compensation system in British Columbia [44], Immigration, Refugees, and Citizenship Canada (IRCC) Permanent Resident database [45], and demographic information from linked BC Ministry of Health Registration data (Consolidation File) [46] to construct a retrospective cohort of workers with a work-related injury requiring at least one day off of work between May 2009 and December 2015. WorkSafeBC operates as a no-fault system, funded through employer-paid insurance premiums, providing coverage to injured workers, including wage-loss benefits, permanent disability benefits, and rehabilitation benefits with the goal of returning workers to work in a timely manner. During the study period, WorkSafeBC provided coverage for approximately 95% of the provincial workforce [47]. The IRCC Permanent Resident database is a repository of people who have been granted permanent resident status in Canada since 1985 and was used to identify workers’ compensation claims for immigrant workers. Data access, extraction and linkage services were provided by Population Data BC, a multi-university data and education resource that supports access to data for research purposes in British Columbia [48]. Ethical approval for the research project was obtained from the Behavioural Research Ethics Board at the University of British Columbia (# H-17-02078).

The study sample was restricted to the first short-term disability (STD) claim per worker in the study period based on four distinct injury cohorts with unique work disability trajectories categorized using the International Classification of Diseases (ICD)-9 codes for injuries. The four injury groups included: (1) Back strain injuries as both an acute and cumulative injury with an episodic recovery; (2) Concussion as an acute injury with an episodic recovery; (3) Connective tissue injuries as a repetitive injury with an episodic recovery; and (4) Upper and lower limb fractures as an acute injury with recovery period. In addition, the cohorts included injured workers aged 15 to 85 years at the time of injury with no missing data on any of the analytic study variables.

### 2.2. Measures

The primary outcome was work disability days defined by the total number of calendar days while on an accepted short-term disability workers’ compensation claim from the date of injury to the date of claim closure, with a follow-up period capped at 365 days. This measure differs from paid wage-replacement days used in other research when detailed return to work (RTW) data are unavailable [49,50] and represents an appropriate measure of the overall burden of work disability.

The primary explanatory variable was immigration classification, defined as economic immigrant, family member immigrant or refugee/other workers based on the immigration record in the IRCC data, or as Canadian-born workers, if there was no record of immigration in the IRCC database.

Potential confounders of the association between immigration classification and work disability included: age at time of injury (15–24, 25–34, 35–44, 45–54, 55+ years), year of injury (from 2009 to 2015), occupation classified into nine groupings according to the 2006 National Occupation Classification [51], annual wage at time of injury measured in five wage quintiles, and history of prior claim (yes/no) in the preceding 5 years (claims data available from 2004 to construct this measure for all included injuries).

### 2.3. Statistical Analysis

Descriptive statistics were used to compare the distribution of the injured worker characteristics across the four injury cohorts. Quantile regression models [52] were used to estimate predicted work disability days for immigrant workers compared to Canadian-born workers at the 25th, 50th and 75th percentiles of the disability days distribution. Quantile regression is an appropriate method when the outcome variable is skewed and allows for the investigation of different effects for those who have longer disability durations as unique sub-groups [53,54]. The models for each injury cohort were adjusted for all confounders and stratified by sex/gender as best practice for health-related research [55,56].

A sensitivity analysis using Cox regression that examined the relationship between immigration classification and time to sustained RTW outcome (return to work, full duties, and with no further disability days) was also performed. This analysis was done to see if there was a difference in effect between work disability days on claim versus time to sustained RTW as two indicators of disability [53]. This approach used piecewise models [57] to calculate hazard ratios (HR) from the first short-term disability day to sustained RTW within 30, 60, 90 and 365 days in order to handle any potential non-proportionality. The models were adjusted for all potential confounders. An HR greater than ‘1’ specifies faster time to sustained RTW or shorter disability duration for an immigrant worker compared to a Canadian-born worker, while an HR less than ‘1’ specifies slower time to sustained RTW or longer disability duration.

All statistical analyses were performed using Stata V.16.0 [58].

## 3. Results

Table 1 presents the distribution of study variables across the four injury cohorts. Approximately 16 percent of all claims in the connective tissue, concussion or upper/lower limb fracture injury cohorts occurred among immigrant workers across the three classifications, while nearly 20 percent of all claims in the back strain injury cohort occurred among immigrant workers across the three classifications. Workers who arrived in Canada as refugee/other immigrants comprised the smallest proportion of claims across all injury cohorts, representing 2.2 percent of claims in the fracture cohort to 2.8 percent in the back strain cohort. Across injury cohorts, the majority of claims were for men; for workers employed in trades and transport or sales and service occupations; and for workers aged 45 to 54 years, although the concussion cohort had a greater proportion of young workers aged 15 to 24 years (20%) compared to other injury cohorts. The frequency of injury claims was relatively stable over time with the exception of the study commencement year, where detailed RTW data was only available from May 2009 onward. The fracture cohort had the lowest occurrence of workers with a prior claim (40%), while the connective tissue cohort had the highest occurrence of workers with a prior claim (54%).

Table 2 presents the results of the fully adjusted quantile regression models with the predicted total work disability days for immigrant and Canadian-born injured workers across injury cohorts and stratified by sex/gender. Overall, the results indicated that, at all points of the distribution of work disability days, with few exceptions, immigrant workers in each of the three classifications had more predicted work disability days compared to Canadian-born workers for back strain, connective tissue, fracture and concussion injuries. The greatest absolute difference in predicted work disability days for immigrant workers across the three classifications with Canadian-born workers was seen in the fracture’s injury cohort, and the least absolute difference was seen in the connective tissue injury cohort.

The largest observed difference in predicted work disability days was seen for workers who immigrated to Canada as a family member or as a refugee/other classification, and, in particular, for women within these classifications, compared to Canadian-born counterparts, at the 25th and 50th percentiles. For example, at the 50th percentile for the concussion cohort, predicted work disability days while on compensation claim benefits for women who immigrated as refugees/other classifications to Canada was 54.2 days (95%CI 24.1, 84.4) compared to 20.1 days (95%CI 13.8, 26.4) for Canadian-born women and compared to 20.6 days (95%CI 11.9, 29.4) for their male counterparts. Similarly, at the 50th percentile for the fracture injury cohort, predicted work disability days on claim benefits for women who immigrated as family members was 94.5 days (95%CI 78.3, 110.7) compared to 67.0 days (95%CI 53.6, 80.3) for Canadian-born women and compared to 78.5 days (95%CI 69.3, 87.8) for their male counterparts. Economic immigrant men and women had comparable predicted work disability days on claim benefits with that of Canadian-born workers across the injury cohorts with few exceptions.

In contrast, gender differences were not as evident at the 75th percentile of the distribution of work disability days, although differences for workers who immigrated to Canada via the family member and refugee/other classifications compared to Canadian-born workers remained.

The smaller sample size of injured workers who arrived in Canada as refugees/other classification resulted in less precise estimates for predicted work disability days as evidenced by wider confidence intervals. Further, there are overlapping confidence intervals, in particular for predicted work disability days for Canadian-born workers with workers who arrived in Canadian via the economic classification, and for immigrant workers who arrived in Canada via the family member classification with those who arrived via the refugee/other classification.

### Robustness of Findings

The Cox regression model to predict sustained RTW after a work disability confirmed a reduced likelihood of RTW for workers in all three immigration classifications compared to Canadian-born workers across injury cohorts, with the largest effect within the first 30 calendar days from the start of work disability (Table 3). The effect remained even after controlling for confounders. For example, in the back strain injury cohort, workers who immigrated to Canada as economic, family member and refugee/other classifications were 12% (HR 0.88; 95%CI 0.85, 0.91), 33% (HR 0.67; 95%CI 0.64, 0.70) and 30% (HR 0.70; 95%CI 0.65, 0.74) less likely to have sustained RTW within 30 days of injury, respectively. While this observed difference in the hazard ratio for sustained return to work diminished over time between Canadian-born workers and workers in all three immigration classifications, a lower likelihood of sustained RTW persisted for refugees/other immigrants within the 90 days and 365-day follow-up windows with few exceptions. Economic immigrants displayed time-varying differences in sustained RTW, with a reduced likelihood of RTW for short (within 30 days) and long durations (90–365 days).

## 4. Discussion

This study investigated differences in work disability duration for workers who immigrated to Canada according to their arrival as economic, family member or refugees/other immigrant classification, and Canadian-born workers, with a workers’ compensation claim for work-related back strain, concussion, connective tissue or upper and lower limb fracture injury in British Columbia, Canada. There are two main findings from the final models. First, workers who immigrated to Canada regardless of their immigration classifications had longer work disability durations (measured as days on work disability claim benefits) than Canadian-born workers across the different injury cohorts and the different points of the disability distribution. Second, disability durations varied by immigration classification and by sex/gender. In particular, workers who immigrated to Canada in the family member and refugee/other classifications, and specifically women within these classifications, experienced the longest disability durations across injury cohorts, while workers who immigrated to Canada in the economic classification had comparable work disability days on claim benefits to Canadian-born workers irrespective of sex/gender. The overlapping confidence intervals around model estimates of predicted work disability days suggests the potential for similar effects or shared experiences/contexts for Canadian-born workers with workers who arrived in Canadian via the economic classification and for immigrant workers who arrived in Canada via the family member classification with those who arrived via the refugee/other classification.

The overall pattern of longer work disability duration among immigrant workers aligns with prior research [6,24,27,28], although the current results of differences by immigration classification illuminate the impacts of different contexts and inequities for immigrant workers and that not all immigrant workers have shared experiences. These contexts, including social, political and structural influences, such as economic policies, programmes and resource distribution approaches, interact and work along gradients of social position, gender and racialization to unevenly benefit some groups over others [59]. Historically, immigrant groups have been conceived as “commodified labour” rather than nation-building citizens [60]. Entry to Canada is demand-driven to meet immediate economic needs, yet the nature of work for many immigrants contributes to underemployment due to deskilling and devaluation of foreign credentials and work experience [61], but also exclusionary practices, such as a lack of access to benefits and paid sick leave for immigrants in high-status occupations [62]. Prior research has found that immigrants end up in jobs where they face excess occupational hazards and lack autonomy and power that may lead to poorer rehabilitation outcomes following an injury [9,32,39]. A number of studies also point to challenges at different stages of the compensation process for immigrant workers that may prolong seeking disability benefits and time off work for injury with implications for recovery, rehabilitation and return to work. These challenges are largely shaped by language barriers [33] and include under-reporting of work-related injuries [22,63,64] and waiting until injuries are more severe to seek compensation, the issue of injury attribution or having their claim recognized as work-related, especially if contested by employers resulting in claim denials and appeals [14,65,66], and provision of appropriate work accommodation [14,22]. The current findings suggest these labour market experiences, and their impacts may be differentially experienced by immigrants who arrive in Canada as refugees or family members; and that arriving in Canada via the economic classification may mitigate some of these experiences and impacts. 

In Canada, the immigration selection system for the economic stream favours individuals with high levels of language, education, work experience, a preferential working age and level of health (e.g., immigrants may be deemed inadmissible if their health condition will endanger public safety or cause excessive demand on health/social services) that facilitates integration to higher skilled and less hazardous jobs. Meanwhile, family member and refugee/other classification immigrants are selected for family reunification purposes and for broader humanitarian reasons, respectively. Post hoc analyses of the study immigration cohort revealed that family member and refugee/other classification immigrants were less likely to have university-level training compared to economic immigrants, and more likely to report their skill level as labourers on arrival to Canada. These two immigration classifications may result in different labour market experiences than for economic immigrants, including for deskilled positions, precarious employment, higher-risk occupations, and smaller workplaces where occupational health and safety practices may be less organized [67,68]. These labour market contexts for family member and refugee/other immigrant workers, all other things being equal in the analytic models, suggests that other factors, such as delayed benefit seeking, more severe injuries, difficulty navigating rehabilitation or return-to-work services or differential treatments for recovery/return-to-work, are contributing to longer work disability durations.

The findings of differences in work disability duration for immigration classification by sex/gender are more complex. Studies that have examined disability duration and sex/gender differences have been mixed [49,53,69], and only one to date has differentiated by immigration status, measured as length of time in Canada [6]. In this one previous study, recent immigrant men (<10 years in Canada) displayed a longer disability duration while recent immigrant women had disability durations closer to Canadian-born women. Although time in Canada since immigration is a valid measure of socioeconomic circumstances and context (e.g., language, work experience may improve the longer one resides in Canada), the observed longer work disability durations for workers who immigrated to Canada as family members and refugees/other immigration classifications in the current study suggests contextual disadvantages at the time of immigration that persists over time and even after integration into the workforce.

When gender intersects with immigration classification, a “perfect storm” of different contexts contributes to longer work disability durations, especially for short- to mid-duration claims. We offer three possible reasons for differences in immigrant women’s longer disability duration compared to their male counterparts. First, prior research has documented that immigrant women experience domestic strain—double workload from employment and fulfilling domestic responsibilities (e.g., childcare, cooking, cleaning) [70,71]. This may result in less time spent on rehabilitation activities to return to work compared to men, leading to longer disability durations [32,70,72]. Cultural representations of gender roles and norms of masculinity, wherein men are considered to be primarily responsible for economic stability, may motivate or pressure immigrant men to return to work earlier than immigrant women [73]. Finally, immigrant women, and, in particular, family and refugee/other classification women, are often recruited to jobs that are socially undervalued and with little opportunity for upward mobility [74] due to stereotypes of these women as fragile and inexperienced [71]. In addition, Canadian immigration policy labels these women as ‘dependent’ to those they arrive with (e.g., as someone’s spouse, daughter, sister) [75]. The circumstances and effects of gendered division in labour, family roles and power structures have worsened immigrant women’s social status in the host country [61] and perpetuated vulnerabilities, granting women fewer rights in the workplace and higher occupational exposures, resulting in potentially more severe injuries requiring longer time off work. However, in the current analyses, gender differences tended to diminish for longer duration claims, as evidenced at the 75th percentile of the work disability distribution. While more research is warranted to understand the gendered findings, injury severity may be a major determinant of long work disability duration, affecting men and women equally, as seen elsewhere [49].

Taken together, our findings suggest that immigration classification is strongly associated with work disability duration and that there are contextual determinants for workers who arrived in Canada as family members or refugees/other immigrant classifications that underpin this relationship. The current research was unable to delineate these contextual determinants. Although, the consistency of the effect of longer work disability durations for family member and refugee/other immigrant classifications, distinct from economic immigrant classification, regardless of the injury cohort (acute, chronic, episodic) and across multivariable models adjusted for socioeconomic, demographic and occupational characteristics, lends credence to the validity of the contribution of underlying contexts to inequities for immigrant workers over the longer term. From a policy perspective, the findings provide a signal to workers’ compensation and occupational health organizations of the potential need for tailored disability management efforts for immigrant workers, specifically family member and refugee/other classification immigrant women. This can be provided in the form of a gradual return to work (temporarily changing a worker’s duties, hours and/or days of work) that has been shown to reduce work disability duration for workers with longer-term claims [53]. Programs and policies that take into account underlying contexts and the drivers of health and safety inequalities for immigrant workers are also needed to facilitate access to and implementation of recovery and rehabilitation services. Family member and refugee/other immigrants often arrive with limited language proficiency as employment integration is not their primary reason for immigration, and evidence suggests that the language needs of these immigrants may not be systematically addressed throughout the return-to-work process [33,76]. Premji and colleagues’ analysis of language accommodation in the jurisdictions of Ontario and Quebec point to the provision of information and services in different languages at no cost to workers’ compensation staff and injured workers as good initial practices [76], while others suggest cultural competency training among health care providers and compensation actors should be implemented over the longer term [32].

The devaluation of foreign education and work experience for many immigrant workers, perhaps especially those arriving as refugees or family members, that contributes to precarious employment relationships, and ultimately the risk of longer disability durations, requires a paradigm shift that is beyond policy at the compensation system level. High-income countries, such as Canada, rely on immigration to sustain population growth and fill crucial labour market shortages, yet immigrants arriving in Canada as refugees or family members are often not afforded the same employment opportunities or career progression as Canadian-born workers or those arriving via the economic classification. Governments, employers and researchers need to examine labour market practices that reduce barriers and biases to promotion and progression in the labour force for all immigrant workers, but in particular for those that arrive in Canada in more vulnerable contexts. Addressing vulnerable contexts that persist over time is an even broader population health and immigration policy issue.

### Strengths and Limitations

A key strength of this study was the access to and linkage of workers’ compensation claim data with immigration records for access to unprecedented population-level data of work disability duration and immigration classification, along with data on known confounders of this relationship. One of the other novel contributions of the research was having a detailed measure of immigration classification that revealed that not all experiences are shared by immigrant workers. The variable measuring immigration classification upon entry to Canada provides a surrogate index measure of the broader determinants of health related to advocacy, equity and context upon arrival to Canada, and that illuminated with differential experiences for workers who arrive in more potentially more vulnerable context via the family member or refugee classification, but not the economic classification, and that had persistent effects even after integration into the workforce over time.

Quantile regression as the main analytic methodology allowed us to examine work disability duration at shorter and longer points of the disability distribution, is appropriate for skewed distributions and is considered an intuitive and interpretable method for direct effect estimates [53].

To our knowledge, this is one of only a handful of studies that links immigration characteristics with workers’ compensation claims data at a population level, offering a novel contribution in terms of approvals from two distinct data stewards (at the provincial and federal level) to access and link their data for research purposes to inform evidence needs on immigration experiences in Canada, including over the longer term and after entering the workforce; and on equity and equality of experiences in the workers’ compensation system.

Despite study novelty and rigour, there are a few limitations to consider. First, reliance on linked administrative data using deterministic and probabilistic linkage procedures may be subject to misclassification when workers with compensation claims (that define the cohorts) are not ‘found’ in the immigration records and are classified as Canadian-born workers. However, the linkage in this study was comparable to other linkages of the Immigration, Refugees and Citizenship Canada database with a provincial health data registry, where 77% of permanent residents with an intended destination of British Columbia were successfully linked to the provincial health registry and an additional 5% with an intended destination elsewhere in Canada were successfully linked. Any bias associated with the misclassification of immigrant workers as Canadian-born workers is hypothesized to have had a conservative effect on the observed findings [77]. There is no evidence to suggest that misclassification of immigrant workers as Canadian-born workers would be differential by immigration classification or work disability duration.

Second, there is the potential for residual confounding from unmeasured variables, such as language proficiency and injury severity, that could bias the association between immigration classification and work disability duration in either direction. Data on measures, such as language proficiency, were not available in linked administrative data for the injury cohorts. We acknowledge that under-reporting of work-related injuries may be higher for those in more precarious employment situations and that this may translate to workers’ compensation claims for more severe injuries in the current study for immigrant workers who report an injury. However, using the Abbreviated Injury Score (AIS), an injury severity score ranging from 1 (minor injury) to 6 (maximal injury) using body region and type of anatomic structure [78], we found minimal variation in severity except in the case of the fracture’s injury cohort with hip and thigh fractures having a severity of 3 versus 2 for all other fractures. Severity could not be measured for the other three cohorts because they were defined by a narrow set of ICD-9 codes with no observed variability in severity using the AIS methods. Inclusion of a severity measure in the current models could have explained some of the longer work disability durations for immigrant workers, and in the absence of a severity measure, immigration classification appears to serve as a surrogate measure, at least in part, for some of the determinants of severity.

As with all epidemiological studies reliant on administrative data, there is the potential for unmeasured confounding. However, we maximized the inclusion of the variables in the model that meet the definition of a confounder for the relationship between immigration classification and work disability duration, including key sociodemographic (age, sex) and socioeconomic variables (wage, occupation), and prior claim status. We argue that other measures, such as access to health care services or navigation of compensation systems, are on the casual pathway and not a confounder for the primary relationship.

Finally, while the model adjusted for prior workers’ compensation claim status in the five years before the study injury, a more detailed post hoc descriptive analysis of prior claims (no prior claim, similar injury claim, different injury claim) by immigration classification revealed that immigrant workers were less likely to have had a prior claim compared to Canadian-born injured workers across all injury cohorts, with the exception that workers who arrived in Canada as family members or refugees/other classifications were more likely to have a similar prior claim (12.8% and 14.7%, respectively) within the connective tissue injury cohort, than Canadian born workers (10.5%). However, collectively across all of the cohorts and the model findings, it does not appear that longer disability durations for immigrant workers who arrived in Canada via family and refugee classifications are readily explained by differential prior injury/claim experiences.

## 5. Conclusions

In this study of injured workers with an accepted compensation claim for acute (fracture and concussion) and chronic injuries (back strain and connective tissue) in the province of British Columbia, Canada, we observed significant differences in work disability durations for workers who arrived in Canada via the family member and refugee/other immigrant classifications, and in particular for women in these classifications, than for Canadian-born workers and immigrant workers who arrived via the economic classification. The findings suggest the need for interventions, such as a provision of gradual return to work for immigrant workers, but also the need for future research that investigates the contextual determinants that underly the longer-term impacts of immigration classification on work disability duration. While the results are in the direction that many researchers and stakeholders might hypothesize, there is value in providing evidence of the relationship between immigration status (as a surrogate measure of the broader determinants of health) and work-related outcomes in order to continue to highlight inequities and vulnerabilities that persist and in order to provide the basis for decision- and policy-makers to argue for changes.

## Figures and Tables

**Table 1:** Sociodemographic and socioeconomic characteristics of injured workers who had an accepted workers’ compensation between 2009 and 2015, by injury cohort.

	Back Strain (*n* = 75,654)	Concussion (*n* = 9489)	Connective Tissue (*n* = 8631)	Fractures (*n* = 12,302)
**Variables**				
**Immigration Classification of Worker ^a^**				
Economic	6666 (8.8%)	726 (7.7%)	638 (7.4%)	871 (7.1%)
Family member	6060 (8.0%)	603 (6.4%)	493 (5.7%)	850 (6.9%)
Refugee/Other	2131 (2.8%)	232 (2.4%)	210 (2.4%)	270 (2.2%)
Canadian-born	60,797 (80.4%)	7928 (83.6%)	7290 (84.5%)	10,311 (83.8%)
**Sex**				
Men	43,770 (57.9%)	5325 (56.1%)	4849 (56.2%)	8701 (70.7%)
Women	31,884 (42.1%)	4164 (43.9%)	3782 (43.8%)	3601 (29.3%)
**Age in years at time of injury**				
15–24	8651 (11.4%)	1939 (20.4%)	939 (10.9%)	1651 (13.4%)
25–34	17,054 (22.5%)	2139 (22.5%)	1681 (19.5%)	2555 (20.8%)
35–44	18,092 (23.9%)	1922 (20.3%)	1967 (22.8%)	2261 (18.4%)
45–54	20,321 (26.9%)	2124 (22.4%)	2714 (31.4%)	3013 (24.5%)
55 and older	11,536 (15.3%)	1365 (14.4%)	1330 (15.4%)	2822 (22.9%)
**Occupation at time of injury**				
Management/Bus	6078 (8.0%)	1010 (10.6%)	585 (6.8%)	1188 (9.7%)
Natural/App. Sciences	1389 (1.8%)	183 (1.9%)	167 (1.9%)	259 (2.1%)
Health	12,076 (16.0%)	553 (5.8%)	976 (11.3%)	455 (3.7%)
Social Sc.	4001 (5.3%)	807 (8.5%)	207 (2.4%)	605 (4.9%)
Art/Culture	949 (1.3%)	297 (3.1%)	79 (0.9%)	272 (2.2%)
Sales/Service	17,918 (23.7%)	2676 (28.2%)	2.312 (26.8%)	2313 (18.8%)
Trades/Transportation	25,919 (34.3%)	2990 (31.5%)	2910 (33.7%)	5410 (44.0%)
Primary	2278 (3.0%)	354 (3.7%)	484 (5.6%)	712 (5.8%)
Manufacturing	5046 (6.7%)	619 (6.5%)	911 (10.6%)	1088 (8.8%)
**Injury Year**				
2009	8229 (10.9%)	651 (6.9%)	900 (10.4%)	1302 (10.6%)
2010	12,437 (16.4%)	1045 (11.0%)	1289 (14.9%)	1888 (15.4%)
2011	12,210 (16.1%)	1263 (13.3%)	1308 (15.2%)	1898 (15.4%)
2012	11,556 (15.3%)	1532 (16.2%)	1317 (15.3%)	1767 (14.4%)
2013	11,039 (15.3%)	1471 (15.5%)	1281 (14.8%)	1753 (14.3%)
2014	10,326 (13.7%)	1694 (17.9%)	1253 (14.5%)	1784 (14.5%)
2015	9857 (13.0%)	1833 (19.3%)	1283 (14.9%)	1910 (15.5%)
**Previous Claim ^b^**				
No	37,376 (49.4%)	5077 (53.5%)	3999 (46.3%)	7444 (60.5%)
Yes	38,278 (50.6%)	4412 (46.5%)	4632 (53.7%)	4858 (39.5%)
**Wage at time of injury**				
1st quintile	14,484 (19.2%)	2500 (26.4%)	1622 (18.8%)	2610 (21.2%)
2nd quintile	15,235 (20.1%)	1926 (20.3%)	1791 (20.8%)	2263 (18.4%)
3rd quintile	15,633 (20.7%)	1639 (17.3%)	1753 (20.3%)	2190 (17.8%)
4th quintile	15,416 (20.4%)	1657 (17.5%)	1778 (20.6%)	2364 (19.2%)
5th quintile	14,886 (19.7%)	1767 (18.6%)	1687 (19.6%)	2875 (23.4%)

^a^ Grouped into four classes depending on the immigration classification upon arrival into Canada. ^b^ Any previous workers’ compensation claim in the past 5 years.

**Table 2:** Predicted work disability days on workers’ compensation claim benefits for workers by immigration classification at the 25th, 50th and 75th percentiles of the disability days distribution by injury cohort, adjusted quantile regression models ^a^.

	**Back Strain (*n* = 75,654)**					
	**25th % (95%CI)**		**50th % (95%CI)**		**75th % (95%CI)**	
**Immigration Classification of Worker ^b^**	**Men** **(*n* = 43,770)**	**Women** **(*n* = 31,884)**	**Men** **(*n* = 43,770)**	**Women** **(*n* = 31,884)**	**Men** **(*n* = 43,770)**	**Women** **(*n* = 31,884)**
Economic	8.4 [7.6,9.1]	9.6 [8.7,10.4]	20.5 [18.4,22.5]	30.1 [26.8,33.3]	53.8 [48.3,59.2]	64.2 [58.7,69.8]
Family member	11.3 [10.4,12.2]	13.0 [11.6,14.4]	33.6 [30.7,36.6]	40.7 [37.0,44.3]	74.4 [68.8,80.0]	80.3 [74.9,85.8]
Refugee/Other	10.4 [9.4,11.5]	13.4 [11.4,15.5]	32.1 [27.7,36.6]	39.5 [34.3,44.8]	78.2 [70.6,85.8]	79.3 [71.1,87.6]
Canadian-born	7.8 [7.3,8.4]	8.4 [7.7,9.2]	19.5 [17.9,21.2]	25.0 [22.2,27.7]	56.1 [51.4,60.8]	63.0 [58.0,68.0]
	**Concussion (*n* = 9489)**					
	**25th % (95%CI)**		**50th % (95%CI)**		**75th % (95%CI)**	
	**Men** **(*n* = 5325)**	**Women** **(*n* = 4164)**	**Men** **(*n* = 5325)**	**Women** **(*n* = 4164)**	**Men** **(*n* = 5325)**	**Women** **(*n* = 4164)**
Economic	5.8 [4.6,7.1]	8.5 [6.6,10.4]	14.7 [9.3,20.1]	21.1 [13.0,29.2]	64.5 [39.0,89.9]	79.5 [52.1,106.0]
Family member	6.7 [5.1,8.4]	9.8 [7.2,12.4]	24.0 [16.0,32.0]	27.7 [18.6,36.0]	100.4 [73.4,127.3]	91.3 [61.9,120.8]
Refugee/Other	6.3 [4.7,7.0]	12.0 [2.9,21.2]	20.6 [11.9,29.4]	54.2 [24.1,84.4]	89.6 [50.0,129.3]	122.6 [76.8,168.4]
Canadian-born	6.2 [5.1,7.2]	6.9 [5.3,8.6]	14.6 [10.4,18.8]	20.1 [13.8,26.4]	62.1 [41.4,82.8]	76.3 [52.0,100.6]
	**Connective Tissue (*n* = 8631)**					
	**25th % (95%CI)**		**50th % (95%CI)**		**75th % (95%CI)**	
	**Men** **(*n* = 4849)**	**Women** **(*n* = 3782)**	**Men** **(*n* = 4849)**	**Women** **(*n* = 3782)**	**Men** **(*n* = 4849)**	**Women** **(*n* = 3782)**
Economic	12.5 [7.2,17.8]	17.9 [10.1,25.7]	33.4 [21.2,45.5]	55.9 [36.5,75.2]	88.0 [57.5,118.5]	120.9 [89.8,152.0]
Family member	13.3 [7.9,18.8]	24.8 [14.6,35.1]	44.4 [31.1,57.7]	66.8 [48.7,84.9]	114.8 [80.2,149.5]	120.3 [91.5,149.1]
Refugee/Other	24.1 [14.7,33.5]	33.3 [15.7,50.5]	53.9 [39.0,68.8]	75.0 [42.4,107.5]	109.9 [80.8,139.1]	150.1 [103.5,196.7]
Canadian-born	12.7 [9.3,16.0]	17.8 [11.1,24.5]	36.6 [27.0,46.2]	59.0 [42.3,75.8]	101.1 [78.7,123.5]	129.9 [102.8,157.1]
	**Fractures (*n* = 12,302)**					
	**25th % (95%CI)**		**50th % (95%CI)**		**75th % (95%CI)**	
	**Men** **(*n* = 8701)**	**Women** **(*n* = 3601)**	**Men** **(*n* = 8701)**	**Women** **(*n* = 3601)**	**Men** **(*n* = 8701)**	**Women** **(*n* = 3601)**
Economic	37.5 [28.8,46.2]	30.5 [19.7,41.3]	71.7 [62.7,80.8]	72.5 [56.8,88.1]	120.9 [106.5,135.3]	120.6 [100.0,141.3]
Family member	41.4 [33.8,49.0]	48.8 [32.9,64.7]	78.5 [69.3,87.8]	94.5 [78.3,110.7]	131.2 [117.4,145.0]	151.4 [121.7,181.0]
Refugee/Other	32.5 [18.5,46.4]	62.1 [45.9,78.2]	80.6 [66.6,94.5]	84.4 [63.9,104.9]	133.4 [107.7,159.0]	125.7 [87.3,164.0]
Canadian-born	31.3 [24.8,37.8]	29.3 [20.0,38.6]	64.6 [57.6,71.6]	67.0 [53.6,80.3]	110.1 [99.0,121.3]	112.3 [94.6,130.0]

^a^ Models adjusted for age, occupation, injury year, previous claim and wage at time of injury. ^b^ Grouped into four classes depending on the immigration classification upon arrival into Canada.

**Table 3:** Likelihood to sustainable return to work for injured workers on a workers’ compensation claim, by immigration classification, injury type and time period from start of work disability benefits ^a^.

	Hazard Ratio (95%CI)			
	**Back Strain Injuries**			
**Immigration Classification of Worker ^b^**	**0–29 days**	**30–59 days**	**60–89 days**	**90–365 days**
Economic	0.88 [0.85,0.91]	1.0 [0.95,1.07]	0.94 [0.86,1.01]	0.76 [0.70,0.83]
Family member	0.67 [0.64,0.70]	0.89 [0.83,0.95]	0.95 [0.89,1.03]	1.00 [0.93,1.08]
Refugees/Other	0.70 [0.65,0.74]	0.86 [0.77,0.95]	0.83 [0.72,0.95]	0.97 [0.86,1.09]
Canadian-born	1.00 (ref)	1.00 (ref)	1.00 (ref)	1.00 (ref)
	**Concussion injuries**			
Economic	0.92 [0.83,1.02]	0.89 [0.68,1.17]	1.06 [0.77,1.47]	0.96 [0.78,1.18]
Family member	0.73 [0.65,0.82]	0.86 [0.66,1.14]	0.88 [0.62,1.26]	0.94 [0.77,1.16]
Refugees/Other	0.72 [0.59,0.87]	0.65 [0.40,1.06]	0.64 [0.34,1.19]	0.79 [0.57,1.10]
Canadian-born	1.00 (ref)	1.00 (ref)	1.00 (ref)	1.00 (ref)
	**Connective tissue injuries**			
Economic	0.92 [0.80,1.05]	1.09 [0.89,1.35]	0.96 [0.74,1.25]	0.85 [0.71,1.00]
Family member	0.76 [0.64,0.89]	1.31 [1.06,1.61]	1.05 [0.79,1.39]	1.01 [0.84,1.22]
Refugees/Other	0.56 [0.42,0.73]	1.18 [0.87,1.61]	0.95 [0.63,1.44]	0.79 [0.59,1.05]
Canadian-born	1.00 (ref)	1.00 (ref)	1.00 (ref)	1.00 (ref)
	**Upper and lower limb fracture injuries**			
Economic	0.87 [0.75,1.01]	0.82 [0.69,0.98]	1.07 [0.91,1.26]	0.85 [0.75,0.98]
Family member	0.63 [0.53,0.74]	0.81 [0.68,0.96]	0.74 [0.61,0.89]	1.04 [0.92,1.17]
Refugees/Other	0.77 [0.58,1.00]	0.62 [0.44,0.87]	0.99 [0.75,1.31]	0.79 [0.63,0.99]
Canadian-born	1.00 (ref)	1.00 (ref)	1.00 (ref)	1.00 (ref)

^a^ Models adjusted for age, occupation, injury year, previous claim and wage at time of injury. ^b^ Grouped into four classes depending on the immigration classification upon arrival into Canada.

## Data Availability

Data were obtained from a third party and are not publicly available. The workers’ compensation claims data, immigration data and demographic data were made available to the researchers by Population Data BC (www.popdata.bc.ca) (data access granted on 13 August 2019) with permission from the data stewards. The data were made available for the sole purpose of achieving the research objectives and are not available for sharing.
